# Unilateral giant axillary accessory breast in male: Case report

**DOI:** 10.1016/j.ijscr.2024.110666

**Published:** 2024-11-28

**Authors:** Ayana Guto Bone, Daba Iticha Ayana, Getahun Jiru Bedada, Tesfaye Birhanu Abebe

**Affiliations:** a Department of Pathology, School of Medicine, Collage of Health Sciences, Salale University, Fiche, Ethiopia; b Department of Surgery, School of Medicine, Collage of Health Sciences, Salale University, Fiche, Ethiopia; c Department of Radiology, School of Medicine, Collage of Health Sciences, Salale University, Fiche, Ethiopia

**Keywords:** Accessory breast, Axillary mass, Histopathology, Lipoma, Male, Case report, 0000, 1111

## Abstract

**Introduction:**

Accessory breast tissue is a rare condition occurring in 1–3 % of males, primarily in the bilateral axillary region. Kajava Class I accessory breast, characterized by glandular tissue, an areola, and a nipple, is rarely reported. This case report highlights the clinical presentation, diagnostic approach, and management of this rare entity in an elderly male.

**Case presentation:**

A 72-year-old male presented with a left axillary swelling since his early twenties. Initially painless and lemon-sized, the mass progressively enlarged, extending over the left chest and flank, causing dis- comfort and restricting daily activities. Physical examination revealed a large, pedunculated mass measuring 25x15x10 cm in the left axilla, with a smooth surface and no skin color changes except in the nipple and areolar region. Diagnostic imaging and cytology confirmed the presence of glandular tissue, and biopsy validated the diagnosis with histologic findings that showed lobules of glandular tissue lined by bland, single-layered ductal and myoepithelial cells, disposed in a fibrous growth background. Additionally, there were foci of bland fat tissue in the histology. Surgical removal of the mass resulted in a successful outcome.

**Clinical discussion:**

The occurrence of Kajava Class I accessory breast tissue in an elderly male is rare. It is important to consider accessory breast tissue in the differential diagnosis of long-standing unilateral axillary swellings. The diagnostic approach included clinical examination, imaging, and histopathology. Surgical excision provided symptomatic relief and pre- vented potential complications such as malignancy.

**Conclusion:**

Kajava Class I accessory breast tissue, though rare, should be considered in elderly males with long-standing unilateral axillary swelling. Early recognition and surgical intervention are crucial for optimal outcomes.

## Highlights

• Accessory breast tissue is a rare condition, occurring in 1-3% of males, primarily in the bilateral axillary region.
• The Kajava Class I accessory breast in elderly male, although rare, should be considered in patients presenting with long standing unilateral axillary swelling.
• Differential diagnoses for axillary swelling include lipoma, fibroadenoma, chest wall tumors, skin lesions, and lymphadenopathy.
• Many patients with axillary breasts opt for surgery due to cosmetic reasons and concerns about potential malignancy.

## 1 Introduction

During embryonic development, if redundant mammary glands do not degenerate as expected, they can form additional mammary glands known as accessory breasts [1,2]. These are usually found along the milk line, which run from the armpits to inguinal regions bilaterally. Accessory breasts are estimated to occur in about 1–6 % of the population, with a higher prevalence in females compared to males with a ratio of 5:1 [3], and more commonly occurring bilaterally rather than unilaterally [4]. This condition is also more commonly reported among the Japanese population [1]. Clinically, accessory breasts can resemble conditions such as lipomas, lymphomas, or various tumors, making accurate diagnosis challenging [5]. This case emphasizes the need to consider accessory breast tissue as a potential diagnosis, even in elderly male patients. Despite recommendations for reassurance and counseling, many patients with axillary breasts prefer surgery due to cosmetic concerns and the fear of malignancy [6]. The combination of polythelia and polymastia in an older male, as in our case, is extremely rare and significant. Given its uncommon occurrence and diagnostic difficulties, it is crucial to report and study such cases. The work has been reported in line with the SCARE criteria [7].

## 2 Case presentation

**Fig. 1:**
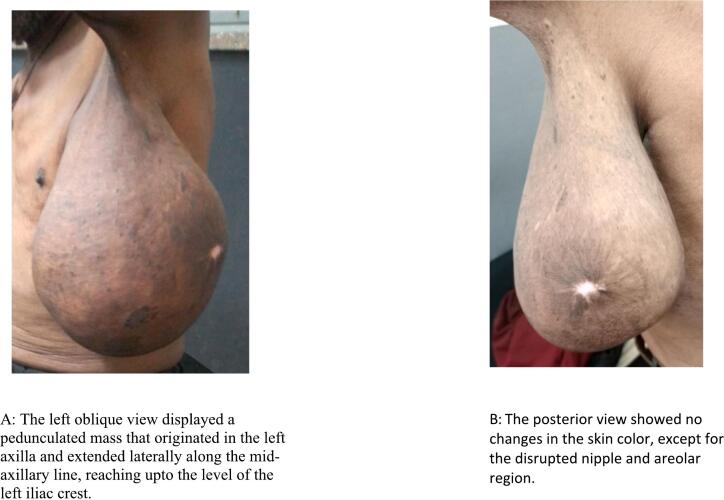
Preoperative photos.

**Fig. 2:**
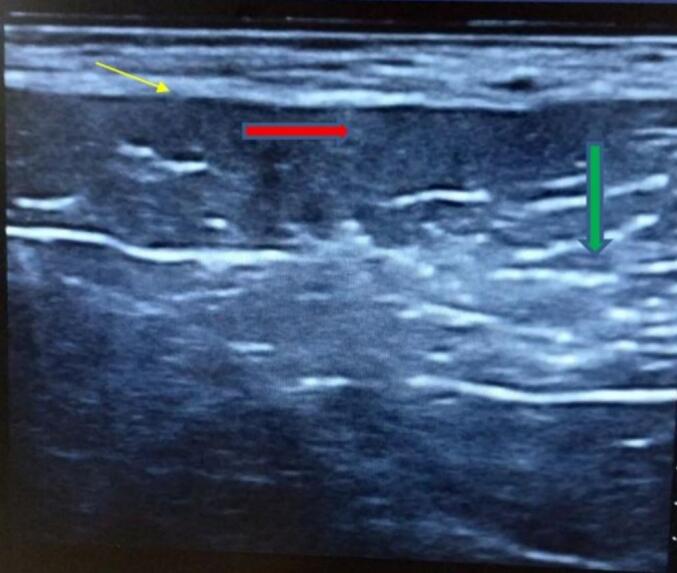
The red arrow demonstrate hypoechoic subcutaneous fat, the green arrow indicate echogenic areas with small hypoechoic regions representing fibroglandular tissue, and the yellow thin arrow show the echogenic superficial fascial layer. (For interpretation of the references to color in this figure legend, the reader is referred to the web version of this article.)

**Fig. 3:**
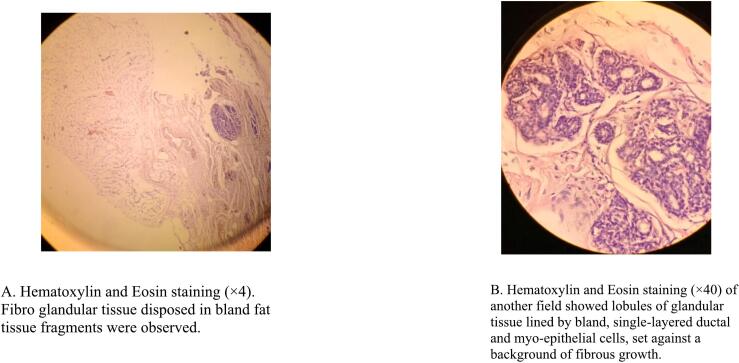
Histological specimen from the axillary mass.

**Fig. 4:**
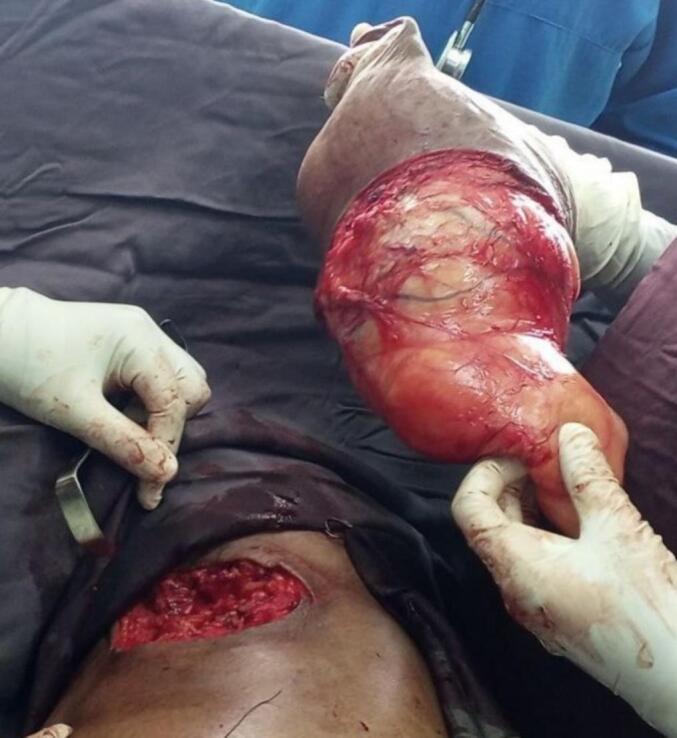
Intraoperative photograph of the excised mass. A giant 24 × 20 × 9 cm mass was extracted.

**Fig. 5:**
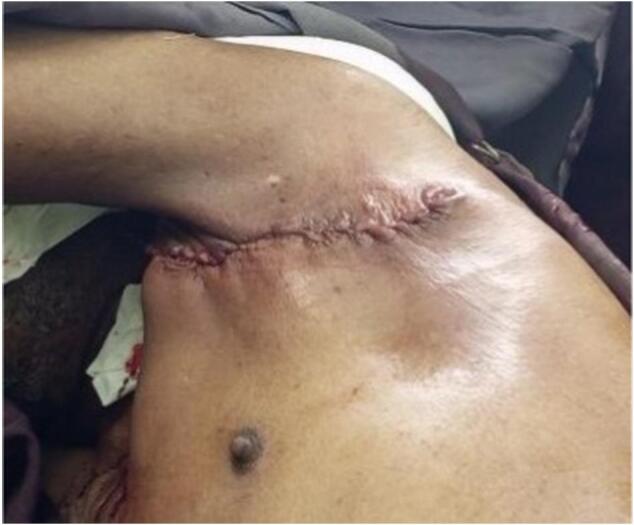
Postoperative photograph. The mass was completely excised with-out damaging the surrounding tissue.

A 72-year-old male patient from rural area was referred to our specialized surgical unit because of swelling in his left armpit, which he had noticed since his early twenties. The swelling began as a painless mass the size of a lemon and gradually enlarged to cover the left side of his chest and flank. This mass caused discomfort and restricted his daily activities, leading him to seek medical help. Although the patient intermittently used traditional medicine on the nipple area of the swelling, this treatment resulted in partial destruction of the nipple and areola instead of reducing the swelling, leaving a pale, scarred area. Aside from the swelling, he did not report any systemic issues or previous surgeries, and he had no other underlying health conditions. On physical examination, a large, stalked, lobulated mass measuring 25x15x10 cm was identified in the left axilla. The mass was soft, non-tender, and had a smooth surface. The skin above it showed no discoloration, except for the nipple and areolar area, which were disrupted. The mass originated in the left axilla and extended laterally along the mid-axillary line to the left iliac crest (Fig. 1). No significant lymphadenopathy was found, and both breasts appeared normal. The patient's vital signs were stable, and no abnormal physical findings were noted aside from those already mentioned. The initial clinical assessment suggested a large left axillary mass, likely related to accessory breast tissue, with a giant pedunculated lipoma as a possible alter- native diagnosis. The patient then underwent an ultrasound and fine needle aspiration cytology, both of which indicated that the mass was accessory breast tissue. The ultrasound displayed a well-defined, uniformly hypoechoic layer of subcutaneous fat with multiple thin, linear echogenic striations and small hypoechoic areas suggestive of fibroglandular tissue (Fig. 2). The patient's complete blood count and electrocardiogram (ECG) were within normal limits. The excised mass was sent for histopathological examination, which confirmed it to be a Kajava Class I accessory breast, characterized by glandular tissue along with an areola and nipple, a condition rarely reported in elderly male in the literature. The macroscopic examination revealed a skin-covered, firm to hard mass with a smooth surface, measuring 24 × 20 × 9 cm. There was a central area of hypopigmented, amputated nipple scar measuring 1 × 1.6 cm. Upon sectioning, the mass was found to be predominantly composed of fatty tissue, with foci of fibrous tissue. Histologic section showed lobules of glandular tissue lined by bland single layered ductal and myo-epithelial cells diposited in fibrous growth back ground. There are foci of bland fat tissue histology (Fig. 3). Based on consistent diagnostic findings from the ultrasound and fine needle aspiration cytology (FNAC) results, the mass was surgically excised under general anesthesia. Intraoperatively, a large pedunculated mass was identified, covering the left lateral chest and flank area. It originated from the axilla and extended laterally to the mid- axillary line, reaching the iliac crest. The mass was well-capsulated and did not invade the thoracic cavity (Fig. 4). The operation proceeded without complications (Fig. 5), and the patient experienced a smooth postoperative course, being discharged on the second day after surgery. He returned to his routine daily activities after the third day of the procedure and was smooth. We followed him for 06 months and Follow-up evaluations showed no signs of recurrence or surgical complications. The patient's clinical presentation, diagnostic approach, and treatment outcomes are summarized in Table 1.

**Table 1:** A summary table that outlines the patient's clinical presentation, diagnostic workup, and outcomes.

Aspects	Details
Clinical presentation	A 72-year-old male presented with swelling in his left armpit that had been present since his early twenties. A large, pedunculated and lobulated mass measuring 25x15x10 cm was identified in the left axilla. The mass was soft, non-tender, and had a smooth surface. No significant lymphadenopathy was detected.
Diagnostic workup	The ultrasound revealed a well-defined, hypoechoic subcutaneous fat layer containing fibroglandular tissue. Lobules of glandular tissue, lined by bland, single-layered ductal and myoepithelial cells, were observed in a fibrous growth background during the histopathologic examination.
Treatment and postoperative outcomes	The mass was surgically excised under general anesthesia. The patient was discharged on the second day and resumed daily activities by the third day. There were no signs of recurrence or complications during the six-month follow-up period.

## 3 Discussion

Accessory breast tissue originates from the mammary ridge, also referred to as the milk streak, during embryonic development [1]. Typically, this ridge regresses, except in the pectoral region where it develops into the standard breast structure. Incomplete regression can lead to the formation of accessory breast tissue along the milk line, which may consist of glandular tissue, nipples, areolas, or a combination of these components [8]. Our case is categorized as Class I according to Kajava's classification system established in 1915, characterized by the presence of glandular tissue along with an areola and nipple [9]. The occurrence of polythelia and polymastia provides important context for understanding accessory breast tissue. While polythelia is generally considered a congenital anomaly, polymastia often arises due to hormonal changes after puberty [8,10]. Accessory breast tissue is reported to affect 2–6 % of females and 1–3 % of males, typically occurring bilaterally in the axillary region, leading to multiple areas of supernumerary tissue growth in approximately one-third of cases [2,11]. Prevalence rates vary significantly by ethnicity and gender, ranging from 0.6 to 5 % among Caucasian and Japanese females, respectively [2]. When compared to similar cases in the literature, accessory breast tissue is often detected incidentally in younger patients, particularly during puberty or pregnancy due to hormonal influence [10,12]. However, our case is exceptionally rare and noteworthy due to the patient's advanced age and the longstanding nature of the mass. The significant size of the mass, along with the history of treatment through traditional medicine instead of modern medical intervention, resulting in scarring, further emphasizes the rarity of this case. The histopathological confirmation of Kajava Class I accessory breast tissue, characterized by glandular tissue and the presence of a nipple, reinforces this uniqueness. The diagnostic approach utilized in this case comprised a comprehensive clinical assessment and imaging studies, including ultrasound. Additionally, fine needle aspiration cytology and histopathological examination were con- ducted. Although MRI is a valuable tool for evaluating soft tissue masses in the axillary region due to its detailed imaging capabilities [13], it was unnecessary in this instance because the ultrasound and FNAC findings provided sufficient diagnostic confidence. Furthermore, while we did not perform immunohistochemistry, considering the case was typical of benign conditions with no clinical suspicion of malignancy and occurred in a resource-limited setting, it is advisable to consider this technique in cases where malignancies such as invasive ductal carcinoma, poorly differentiated adenocarcinoma, liposarcoma, rhabdomyosarcoma, and lymphoma are suspected [6,14]. Surgical excision was performed not only to alleviate the patient's symptoms but also to mitigate potential complications such as infection, ulceration (considering the large and vulnerable nature of the mass), and malignancy. Although accessory breast cancer is a rare form of breast cancer, typically found in the axillary or inguinal regions where lymph nodes and capillaries are abundant, with an incidence rate of 0.3–0.6 % [15], this surgical intervention ensured a comprehensive and effective treatment outcome.

## 4 Conclusion

This case underscores the significance of considering accessory breast tis- sue as a potential diagnosis, even in elderly male patients who present with unilateral axillary swelling of longstanding duration. Early diagnosis, coupled with promoting health-seeking behavior within the community and timely surgical intervention, not only alleviates symptoms but also prevents potential complications.

## Registration of research studies

No first in man case report was published by the authors.

## CRediT authorship contribution statement

Daba Iticha Ayana: Writing– original draft of the case presentation, Data curation, Resources, Conceptualization.

Ayana Guto Bone: Writing– Data curation, Resources, Conceptualization.

Tesfaye Birhanu Abebe: Writing– original draft of the case, review & editing, Visualization. Getahun Jiru Bedada: Writing– review & editing, Resources, Data curation, Supervision.

## Consent

Written informed consent was obtained from the patient for publication and any accompanying images. A copy of the written consent is available for review by the Editor-in-Chief of this journal on request.

## Ethical approval

Approval for this case report was secured from the Ethics Committee of the institution under Approval Number: 0073/2024. The patient has provided written informed consent for the publication of their case details and any related images.

## Guarantor

Tesfaye Birhanu Abebe, the corresponding author, is the guarantor.

## Patient perspective

I had a large lump under my left armpit that led me to withdraw from social life and always wear long-sleeved clothes. I first noticed it in my early twenties when it was small, but it gradually grew over decades to cover my side. Initially, I ignored it, hoping it would go away on its own. I even tried traditional herbal medicines on the nipple area, but nothing improved. Fearing it might be cancerous, I visited the local health center and was referred to a larger hospital. I don't have any similar illnesses in my family, so I was unsure how I got it. I was relieved when I learned that the swelling wasn't cancerous. I'm grateful to be free of such a large lump, and my left arm is finally “out of jail.”

## Sources of funding

No funding was received.

## Funding

No funding was received.

## Declaration of competing interest

The authors declare that they have no conflict of interest.
